# Ambulance Commanders’ Reluctance to Enter Road Tunnels in Simulated Incidents and the Effects of a Tunnel-Specific e-Learning Course on Decision-Making: Web-Based Randomized Controlled Trial

**DOI:** 10.2196/58542

**Published:** 2025-03-28

**Authors:** Johan Hylander, Lina Gyllencreutz, Michael Haney, Anton Westman

**Affiliations:** 1 Department of Diagnostics and Intervention, Surgery Umeå University Umeå Sweden; 2 Department of Nursing Umeå University Umeå Sweden; 3 Department of Diagnostics and Intervention, Anesthesiology and Intensive Care Medicine Umeå University Umeå Sweden; 4 Department of Anaesthesia and Intensive Care Medicine Karolinska University Hospital Huddinge Stockholm Sweden

**Keywords:** e-learning, major incident, incident management, disaster medicine, road tunnels

## Abstract

**Background:**

The optimal response to a major incident in a road tunnel involves efficient decision-making among the responding emergency services (fire and rescue services, police, and ambulances). The infrequent occurrence of road tunnel incidents may entail unfamiliarity with the tunnel environment and lead to uncertain and inefficient decision-making among emergency services commanders. Ambulance commanders have requested tunnel-specific learning materials to improve their preparedness.

**Objective:**

We aimed to assess decision-making among ambulance commanders in simulated road tunnel incidents after they had participated in a tunnel-specific e-learning course designed to support timely and correct decisions in this context.

**Methods:**

We conducted a web-based intervention study involving 20 participants from emergency medical services in Sweden who were randomly allocated to a test or control group. The control group (n=10, 50%) received a lecture on general incident management, while the intervention group (n=10, 50%) completed an e-learning course consisting of 5 modules focused on tunnel structure, safety, and collaboration in response. The participants took part in 2 simulation-based assessments for ambulance commander decision-making in major road tunnel incidents 1 month and 6 months after their allocated study intervention. In each simulation, the participants decided on the best course of action at 15 independent decision points, designed as multiple-choice questions. The primary outcome was the correct response to the question regarding how to appropriately enter the road tunnel. The secondary outcome measurements were correct or incorrect responses and the time taken to decide for each of the 15 decisions. Limited in-depth follow-up interviews were conducted with participants (n=5, 25%), and collected data were analyzed using qualitative content analysis.

**Results:**

All 20 participants completed the first simulation, and 16 (80%) completed the second. The main finding was that none (0/20, 0%) of the participants correctly answered the question on entering the tunnel system in the 1-month assessment. There were no significant differences between the groups (*P*=.59; 2-sample test of proportions) in the second assessment. The e-learning course was not associated with more correct answers at the first assessment, including accounting for participant factors (mean difference between groups: –0.58 points, 95% CI –1.88 to 0.73; *P*=.36). The e-learning course was also not associated with a shorter time to completion compared to the nonintervention group in either assessment. Interviews identified 3 categories linked to the main outcome: *information* (lack of), *risk* (limited knowledge and equipment), and *mitigation* (access to maps and aide-mémoire).

**Conclusions:**

Participation in a tunnel-specific e-learning course did not result in a measurable change in ambulance commanders’ decision-making behavior during simulated road tunnel incidents. The observed hesitation to enter the road tunnel system may have several plausible causes, such as the lack of actionable intelligence and tunnel-specific plans. This novel approach to assessing commander decision-making may be transferable to other educational settings.

## Introduction

### Background

The United Nations projects that 68% of the world’s population will live in urban areas by 2050, with an estimated increase from 4.2 billion to 6.7 billion people [1]. Increased urbanization and growing cities will require reliable infrastructure [2]. Traffic congestion is expensive for both communities and individuals. The analytical company INRIX reported that traffic congestion costs the average American 97 hours and US $1348 annually [3]. To limit traffic congestion and exhaust emissions, road tunnels are key to future urban development [2,4]. Traffic incidents in road tunnels may result in significant societal costs and individual health impairment.

Moreover, increasingly complex road tunnels are being constructed. For example, the Norwegian Rogfast ferry replacement project, which is 26.7 km long and 392 m deep, will become the world’s longest and deepest subsea road tunnel upon completion in the early 2030s [5]. Furthermore, road tunnels can have different designs, including single or separate twin-tunnel tubes. Some tunnel systems include subsea passages and multiple on-ramp and off-ramp systems. In addition, networks of different tunnels may include emergency exits and rooms for technical equipment, shelters, and service tunnels [6]. Understanding the layout of a specific tunnel may benefit emergency services and maintenance personnel responding to a road tunnel incident.

When a major incident occurs in a road tunnel, such as a fire or a traffic crash, the evacuation of tunnel users is the first step in limiting casualties. A complex tunnel structure may hamper self-evacuation and complicate the accessibility of the responding emergency services (police, fire and rescue, and emergency medical services [EMSs]) to the incident site and injured individuals. Moreover, the compact designs of modern road tunnels contribute to unique medical challenges concerning specific injury patterns, specific trauma mechanisms, and increased extraction times [7-9]. Hence, accessibility issues may result in considerable delays in vital treatment, potentially leading to preventable loss of life.

Timely and adequate decision-making by emergency service commanders to facilitate correct task priority and minimize time to treatment is crucial and difficult. This is illustrated by the 2013 Norwegian Gudvanga monotube tunnel fire, where a decision was made to ventilate smoke to aid firefighter access, which, in turn, engulfed 67 evacuating persons in smoke, resulting in acute smoke inhalation injuries [10].

A study by Kristiansen et al [11] exploring the collaboration between responding emergency service commanders during a Norwegian road tunnel exercise concluded that the ambulance commanders were treated as outsiders in the decision-making process owing to uncertainty regarding their management roles. Similarly, in Sweden, an imbalance between the incident commanders and ambulance or medical commanders concerning specific road tunnel knowledge has been identified as a collaborative obstacle [12]. As important decisions need to be taken within the initial 20 minutes of the incident, this obstacle may lead to further delay [13]. The current prehospital medical management system in Sweden uses a general all-hazard approach. This means that the same management method is used regardless of the incident type or environment [14,15]. In contrast, Norwegian fire and rescue services include educational material on decision-making, task allocation, risks, human behavior, and coping with uncertainties in their training curriculum [16]. Swedish ambulance staff with road tunnels in their catchment areas are expected to initiate incident management in the event of a road tunnel incident, even without tunnel-specific knowledge.

Knowledge of the tunnel system and the ability to understand emerging situations are important for fire and rescue service commanders and personnel when responding to road tunnel fires [17]. Studies concerning EMS incident management in road tunnel incidents in Norway [18] and Sweden [19] have found that there is a perceived need for tunnel-specific and readily accessible learning materials and training courses. These are needed to be better prepared to optimally assess response needs in a complex environment and make timely decisions to facilitate early access to the injured persons.

### Objective and Hypotheses

The main aim of this study was to evaluate whether participation in a tunnel-specific e-learning course affected ambulance commanders’ abilities to make appropriate decisions in simulated road tunnel incidents. The secondary aim was to explore participant confidence in decision-making through structured questions and responses.

#### Hypothesis 1

Hypothesis 1 is that the tunnel-specific e-learning course will lead to more participants correctly choosing to enter the road tunnel system in the 1-month postcourse simulation-based assessment compared to those who have not participated in the course. Furthermore, the same is hypothesized for the second assessment, 6 months after the e-learning course intervention.

#### Hypothesis 2

Hypothesis 2 is that the tunnel-specific e-learning course will lead to participants giving more correct answers across the 15 decision points for the simulated tunnel incidents (fire or car crash) compared to those who have not participated in the course, taking into account relevant participant factors, including educational level and previous practical experience with tunnel incidents.

#### Secondary Hypothesis

The secondary hypothesis is that tunnel-specific e-learning course participation will lead to a shorter time to make decisions in the simulation-based assessments compared with no e-learning course participation.

## Methods

### Study Design

**Figure 1:**
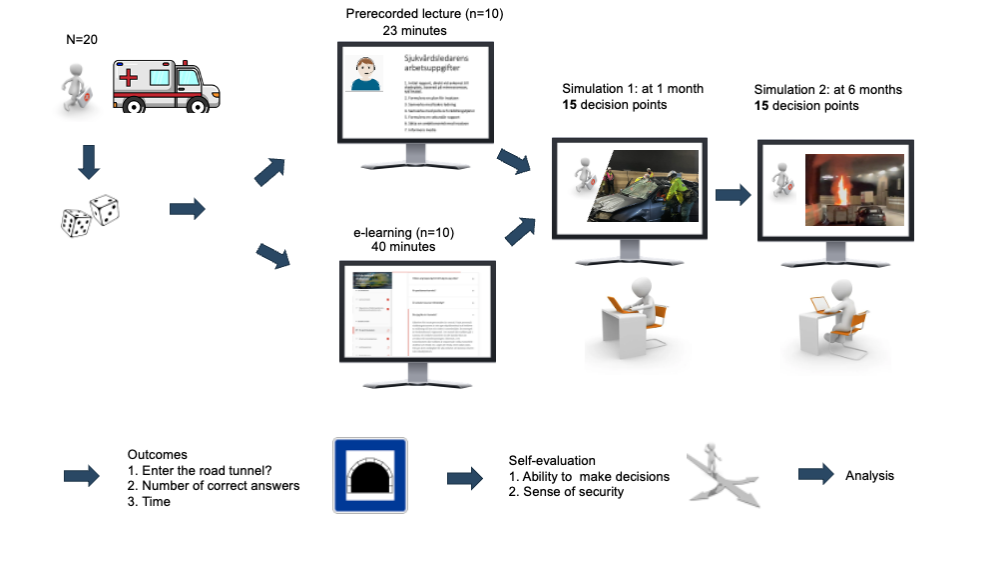
Schematic overview of the study design. This study is a 2-arm, assessor-blinded, randomized, prospectively controlled trial. Participants (N=20) were enrolled and randomized to either a prerecorded lecture or the tunnel-specific e-learning course. Both groups completed follow-up simulations at 1 and 6 months, which included 15 decision points (each designed as a multiple-choice question). Outcomes included the decision to correctly enter the tunnel, the number of correct decisions, and time. Participants also self-evaluated their decision-making ability and sense of security in handling road tunnel incidents.

The difficulty in evaluating the effectiveness of educational efforts in the medical management of major incidents has been described in the literature [20]. This study has taken an innovative approach to this issue. A schematic overview of the study design is presented in Figure 1. The design of this study is a 2-arm, assessor-blinded, randomized, prospectively controlled trial. The intervention was web based and accessed by individual participants via a purpose-built online platform. This study adhered to the CONSORT-EHEALTH (Consolidated Standards of Reporting Trials of Electronic and Mobile Health Applications and Online Telehealth) reporting guidelines [21,22].

### Ethical Considerations

This study was approved by the Swedish Ethical Review Authority (registration numbers 2021-04810 and 2022-05388-02) and conducted in accordance with the Declaration of Helsinki [23]. As this study examined the effect of an educational course on health care provider performance in a simulation, trial registration was not deemed necessary, as described by the International Committee of Medical Journal Editors recommendations [24]. Potential participants were provided with written information, including informed consent descriptions, regarding the aim of the study and the voluntary nature of participation. They were also informed that deidentified data (eg, demographic data, such as sex and educational level) would be included in a scientific article. In addition, they were given the contact information of the study’s data protection officer if, after enrollment, they wished to have their personal information removed. Potential participants were also informed that they would not receive any compensation for participation. Informed consent was obtained from the participants before they were given access to their allotted course material (refer to the Study Sequence section and Multimedia Appendix 1 for further details).

### Setting

This study was conducted between March and December 2022, with participants from a large city in southern Sweden that has several twin-tube road tunnels in its catchment area. In the studied city, the EMS comprised emergency ambulances, physician-staffed vehicles, a dedicated incident management unit, and a helicopter. Only staff from the dedicated incident management unit and emergency ambulances were included as study participants. These units were staffed by either a registered nurse and an emergency medical technician or 2 nurses. The educational level of those who staff emergency ambulances in Sweden varies from emergency medical technicians to registered nurses with a 1-year subspecialization, such as prehospital care. The EMS personnel are trained in advanced life support and prehospital management leadership. The prehospital management leadership course provided for Swedish EMS staff is a mandatory 2-day course covering the basics of incident management and draws from the Major Incident Medical Management and Support (MIMMS) course [14,25]. A separate 2-day leadership course focusing on stress resilience and leadership traits is available, although this course is not mandatory or nationally implemented [15].

In Sweden, including the studied region, the standard operating procedure (SOP) concerning major incidents is that the first arriving ambulance crew at an incident site initiates “command and control” procedures by assuming the roles of ambulance and medical commanders [25]. Consequently, all personnel who staff emergency ambulances need to be able to initiate command and control.

### Sample Size Calculation, Participants, and Randomization

#### Sample Size Calculation

The sample size calculation was based on findings from local pretesting and recent literature [18,19], where ambulance commanders highlighted concerns about tunnel risks that could lead to hesitation in entering the road tunnel system. On the basis of this, the control group was expected to respond with close to 0 (10% response rate) correct answers to the critical categorical question (decision to correctly enter the road tunnel) and, similarly, a close to 0 (10%) correct response rate to the 15 tunnel-specific decision points (given their lack of tunnel-specific education and unfamiliarity with the tunnel environment). The intervention group was expected to correctly answer the critical single categorical question and to have a general correct response rate of 75% for all questions. Using a power of 0.8 and a 2-tailed α of .05 with the Pearson chi-square test, the estimated sample size was 8 participants per group. We aimed to include a minimum of 10 participants in each group owing to anticipated dropouts after inclusion.

#### Inclusion and Exclusion Criteria

The inclusion criteria were participants who were currently employed by the regional EMS and assigned to the ambulance service or dedicated incident management unit as either registered nurses or emergency medical technicians. The participants could also assume the role of ambulance commander in road tunnel incidents, in accordance with the SOP. The exclusion criteria were individuals who were involved in the development or validation of the tunnel-specific e-learning course for this study and those who were diagnosed with or had self-perceived posttraumatic stress disorder or anxiety due to road tunnel incidents.

#### Recruitment Process

The ambulance station commander assisted in informing potential participants of the regional study. A letter of invitation to participate in the study was distributed via “a weekly newsletter” in the organization. Furthermore, short presentations concerning the aim of and participation in the study were conducted by the first author during the organization’s morning briefings. In total, 23 participants were recruited during a 3-month period (March to May 2022), of whom 20 (87%) participants were included in the study, while 3 (13%) were in reserve. Before the intervention was initiated, 3 (13%) planned participants withdrew, and the 3 (13%) individuals in reserve were included as participants.

#### Randomization

The first author collected the participants’ email addresses. The 20 participants’ email addresses were sorted in alphabetical order and assigned a case number. The participants were randomly allocated to the intervention or control arm using a random number generator. A randomization key containing case identification details was stored in a locked place to which only the first author had access.

### Course Material and Study Sequence

#### Online Platform

An online platform was created for this study using Moodle (Moodle HQ) open-source software [26]. The online platform contained both the e-learning course and the control (nonintervention) “course.” Participants were given individual access to one of the courses after a randomized allocation. Furthermore, both simulations were stored on the platform (hidden from the participants) and became available during the assessment periods (1 and 6 months after the educational intervention). Once started, neither simulation could be aborted. Thus, the participants had 1 opportunity to complete each simulation. The participants were informed of this multiple times in writing.

#### Prerecorded Lecture (Control Arm)

The control course consisted of a 23-minute prerecorded lecture on general incident management. The lecture consisted of a brief historical overview of major incidents where incident management was highlighted as important, including examples from the 2011 bombing in Oslo, Utøya island shooting in Norway, and the 2017 truck attack in Stockholm, Sweden [27,28]. The lecture emphasized the roles of the first ambulance at the incident site, focusing on the ambulance commander–specific tasks according to the MIMMS concept, with emphasis on the mnemonic METHANE (major incident, exact location, type of incident, hazards, access and egress, number of casualties, and emergency services) [25]. The use of the mnemonic METHANE has been an SOP in Swedish EMS prehospital incident management for the past 20 years [29]. As such, the general lecture did not include new learning or tunnel-specific elements. The purpose of the control course was to refresh the participants’ general knowledge of incident management in major incidents. The control course was validated by 5 EMS personnel who had extensive prehospital experience from parts of the country outside the studied region. The personnel were asked to provide feedback on the content of the course and perceived usefulness to ambulance commanders. Feedback included comments, for example, “Please describe MIMMS a bit more, otherwise the lecture contains much that I already knew.” Changes were made according to their feedback.

#### The Tunnel-Specific e-Learning Course (Intervention Arm)

The intervention in this study consists of a tunnel-specific and gamified e-learning course created using Articulate Global software [30]. The e-learning course was developed in collaboration with the Faculty of Humanities at Umeå University, Sweden.

The aim of the e-learning course was to increase ambulance commanders’ ability to make the proper decision at the right time by increasing knowledge of the specific tunnel environment. A particular concern was giving participants the confidence to enter the road tunnel in a safe manner, for example, using the unaffected tunnel tube during a major incident, which, according to discussions among stakeholders, is an effective method for gaining access to the scene and those injured.

The e-learning course (content outline is provided in Multimedia Appendix 2) consisted of materials concerning collaboration in a tunnel-specific context and organizations’ different tasks from dispatch to arrival at the incident scene [18,19,31], methods of identifying injured individuals (eg, triage), principles for treating injuries (eg, smoke inhalation), and specific risks (eg, explosions) [32-34]. The e-learning course content was validated by 8 stakeholders from organizations involved in road tunnel rescue efforts, including fire and rescue services, EMS, police, emergency dispatch centers, and infrastructure owners. One or 2 participants from each organization participated. In addition, the course content was reviewed by 2 individuals with in-depth knowledge of prehospital education and extensive experience in acting as ambulance commanders in a variety of scenarios. Changes were made according to their feedback.

The e-learning course consisted of 5 distinct modules with learning materials on different aspects of road tunnel rescue efforts, including the road tunnel structure, potential injury patterns, safety, and collaboration, which were determined after the validation process. The modules were, in collaboration with an expert in pedagogy, incorporated into a gamified e-learning setting. This design was deemed suitable as an element of gameplay that may stimulate learning [35]. The learning materials consisted of text, images (eg, illustrating how to correctly position an ambulance in a major road tunnel incident), and short videos (eg, showing an example of fire development in a road tunnel fire) combined with module-specific quizzes (respondents needed to score 80% correct answers to pass each module) to facilitate learning [36] (refer to Multimedia Appendix 3 for an example).

Road tunnel layouts, different injury patterns, collaboration, and aspects of personal and scene safety were integrated into the e-learning course modules. Using the revised version of the taxonomy of learning by Bloom, which was proposed by Krathwohl [37], study participants needed to *understand* (eg, the layout of the tunnel, tunnel-specific risks, and probable injury patterns), *analyze* (how to collaborate with other organizations to get a clearer picture of the incident and incident-specific risks), and *evaluate* (the tunnel layout, risks, and specific injury patterns) to be able to make an informed decision regarding where the injured person should be transported for definitive care. As decision-making is a complex cognitive process, different aspects were covered in the e-learning course. For example, risk management included the identification of environmental risks (smoke and leakage of flammable or corrosive fluids) and personal risks (inhalation of toxic fumes and limited visibility in a smoke-filled environment). Furthermore, risk-mitigation measures (eg, how to position ambulances in the tunnel system) and tunnel safety features (fire suppression systems and jet fans) were described. Under the course segment “Should I enter the road tunnel?” participants were informed of the importance of conducting a risk assessment before entering the road tunnel system and that using the unaffected tunnel tube (in case of a fire or major incident) was considered safe.

The completed e-learning course was validated by 5 EMS staff members (who also validated the control course) based on language, logical progression, design, and overall usability. The overall impression of the course design was described as “appealing.” The course was described as “easy to navigate” and “fun,” although “not designed to be used via smartphone.” Changes were made according to validating EMS staff members feedback; for example, those included in the study were advised not to use smartphones while taking their allotted courses or simulations.

#### Prospectively Controlled Trial

The trial consisted of a knowledge test based on the tunnel-specific intervention’s course content, which was tested in 2 digital simulations (assessment points) conducted 1 month and 6 months after the intervention. The simulations also included a time measurement for decision-making. Time was measured individually for the 15 separate decisions in each simulation. Between the first and second knowledge tests (assessment points), there was no control over for what the participants read, studied, or discussed in relation to tunnel incident responses.

#### Knowledge Test

The knowledge test was developed based on the literature on performance indicators [38-40], interview findings [18,19,31], and focus group discussions among stakeholders [13]. A total of 15 independent multiple-choice questions (MCQs), each with 1 correct option out of 5 alternatives, were created. This design was chosen because MCQs are commonly used in medical education to assess higher-order thinking, so the participating personnel should be accustomed to the method [41]. In total, the participants could get 15 points per simulation. The questions were formulated as decision points (Multimedia Appendix 4) and were directly related to the content of the intervention (e-learning course). The questions were evaluated during the pilot testing of the digital simulations.

#### Simulations

**Figure 2:**
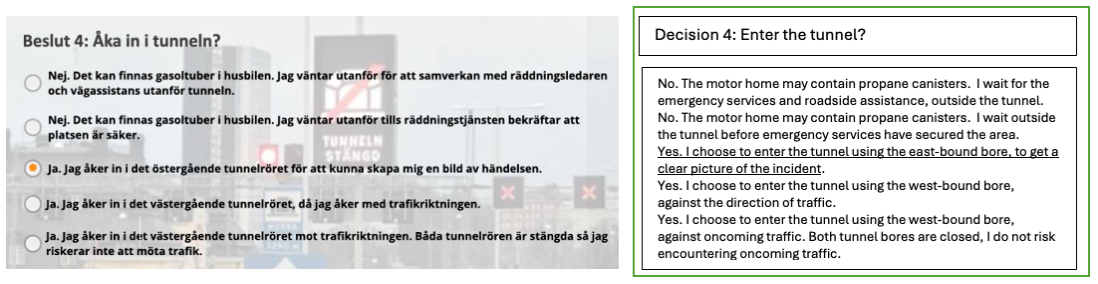
Decision from one of the simulations on how to enter the road tunnel, designed as a multiple-choice question with 5 different choices, one of which is correct. The text is provided in Swedish, with the corresponding English translation shown to the right (the correct answer is underlined).

The digital simulations were created in collaboration with an expert in pedagogy and by integrating learning materials into an e-learning environment. The content was created using Microsoft PowerPoint (version 16.71) and modified using Storyline 360 (Articulate Global, LLC). The 2 simulations of major road tunnel incidents were visualized through a combination of text, images, and videos (eg, exercises); audio (dispatch calls and alarm signals); and animations to add a sense of realism. To mimic a real tunnel incident, the simulations followed the same logical, decision-based pathway, starting from dispatch and continuing through to the conclusion of a rescue effort. One example of a decision-based question is “Do you enter the road tunnel”? (Figure 2).

The simulations were designed as forward-moving only. The aforementioned kind of modified essay question is commonly used in medical education, where students are presented with a case and gradually presented with more information as the case progresses, similar to unfolding an incident where initial information may be scarce [42]. The scenario unfolds regardless of what the participant answers, and the participant cannot go back and change answers to the previous questions. During the simulation, participants received updated information via an audio clip (eg, prerecorded dispatch call) or an image containing information on certain decisions made by others, for example, “the road traffic center decided to close the road tunnel.” Actual footage or video sequences from road tunnel interiors were also included.

Two different simulations (1 month and 6 months after the educational intervention) were created. In the first assessment (at 1 month), the simulated road tunnel incident was a collision between a bus and a passenger car, with numerous casualties. The scenario did not include fire; smoke; or added risks, such as leakage of fuels or dangerous cargo. In the second assessment (at 6 months), the scenario was a collision between a mobile home and multiple vehicles, again with numerous casualties. The mobile home contained a liquefied petroleum gas cylinder as an added risk element. This scenario developed into a road tunnel fire at a later stage. These 2 scenarios were chosen based on the experience that most fatalities in tunnel incidents result from traffic crashes and fire [43].

The simulations were validated separately by 5 EMS personnel (external validation) and 2 stakeholders (internal validation) who had in-depth education in prehospital management and extensive experience in acting as ambulance commanders. Their feedback included comments such as “The time pressure was noticeable, I chose to read instead of listen...I lost focus...But the same goes for a real incident.” After the feedback was considered and changes were made to the simulations, a final version was established by the coauthors. No changes were made to either course once data collection from study participants was started.

Besides the choice of decision in each simulation, the time taken to make each decision was also recorded. This was a feature available in the software used in the development of the simulations (Storyline 360). When the participant was presented with the MCQ options, a hidden timer started, and the timer stopped when the participant chose an alternative and clicked on “next” to advance to the next question. The timing for each question was validated separately by the first author and the e-learning expert using stopwatches. Time was also measured during the evaluation of the simulations.

#### Study Sequence

Individual emails containing information about the study, together with a unique link to either the control or intervention course, were sent to the participants. When the participants clicked on the link, they were directed to a website (where the course platform was located) and prompted to log in. After logging in and before gaining access to their allotted course, the participants were asked to answer a short survey regarding their demographics: sex, level of education (registered nurse or emergency medical technician), additional 2-day leadership course, the number of years in EMS, and experience in managing tunnel incidents. Furthermore, the participant had to give consent by ticking a checkbox before completing the survey. Upon completion of the survey, participants could open their respective courses and had 2 weeks to access the content and complete the course.

Approximately 1 month after the participants completed their respective courses, they were sent an email with an invitation to log onto the e-learning website to complete a simulation and assessment, again with the expectation of participating within 2 weeks. As described earlier, the scenario contained 15 decision points where the participant could decide on the best course of action, choosing from multiple choices, with 1 correct answer. The participants’ responses and the time taken to make each decision were recorded.

Six months after the participants completed their respective courses, they were sent an email prompting them to log in to the e-learning website to complete the second simulation and assessment (scenario details given earlier) and again within 2 weeks. Participants who had provided their phone numbers were sent an SMS text message reminder. The participants’ decision responses were recorded in the same manner.

#### Evaluation Form

Upon completion of the second simulation, the participants were redirected to an evaluation form and asked to subjectively assess (decreased, unchanged, or increased) whether the course (intervention or control) influenced their ability to make decisions and affected their sense of security as ambulance commanders in road tunnel incidents. In total, 2 email reminders of participation were sent after 5 days and 10 days. The data collection period was extended by 1 week because of a limited response rate, and the time extension yielded 5 additional responses.

#### Follow-Up Interviews

A limited number of in-depth follow-up interviews were conducted with participants (4/10, 40% from the intervention group and 1/10, 10% from the control group) within 2 months from the second simulation. The interviews were conducted using a semistructured interview guide and focused on participants describing their reasoning behind the decision to enter or not enter the road tunnel in both scenarios (Multimedia Appendix 5). The collected data were analyzed using the method of qualitative content analysis [44], where the transcribed data were broken down (deconstructed) into meaning units. Next, each meaning unit was labeled with a code, and similar codes were grouped into subcategories and categories.

### Outcomes and Analysis Plan

#### Outcomes

The outcomes were defined as choosing the correct decision when presented with MCQs and the time spent making each decision. The first primary outcome was the correctness of responses to the question concerning the ambulance commander’s decision to enter the road tunnel. The second primary outcome was the number of correct responses across the 15 different decision points. The secondary outcome was the time taken to register each decision.

After the conclusion of the simulation-based assessments, participants were asked to self-evaluate whether they felt that the e-learning course or control course influenced their ability to make decisions in general in road tunnel incidents (decreased, unchanged, or increased) as well as influenced their sense of safety in acting as ambulance commanders in road tunnel incidents (decreased, unchanged, or increased).

#### Data Curation and Availability

Pseudonymized participant test scores and response times were electronically recorded. The dataset was stored on an encrypted hard drive (2 TB; ADATA Technology Co, Ltd).

#### Statistical Analysis

Statistical analysis was performed using SPSS Statistics software (Macintosh version 28; IBM Corp). A 2-sample proportion test was used to compare the grouped averages of proportions. *P*<.05 was considered significant for identifying the difference between groups. Multivariate logistic regression was used to assess the associations between the primary outcome and participant factors as well as between secondary outcomes and participant factors.

## Results

### Participants

**Figure 3:**
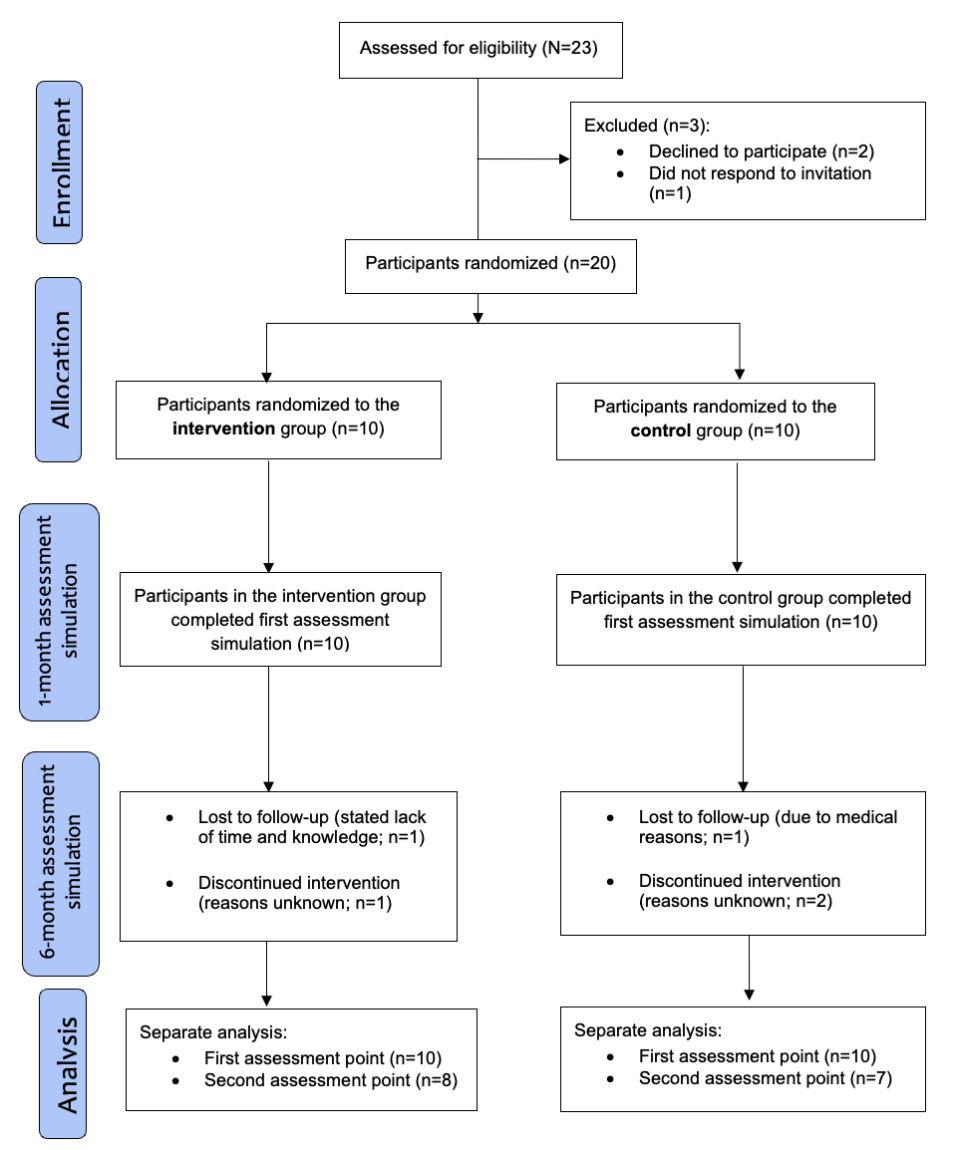
Study flowchart. Description of the number of participants enrolled and lost to follow-up during the 6-month study period.

A total of 20 participants participated in the course to which they were assigned and the first simulation-based assessment. Of these, 16 (80%) participants, with 8 (40%) in each group, participated in the second simulation-based assessment at 6 months. However, 1 (5%) participant in the control group did not complete the assessment, and data from this participant were not included in the grouped responses for the second assessment (Figure 3). Participant demographics, such as sex, years of experience, and educational level, are provided in Table 1.

**Table 1:** Participant demographics, including sex, years of experience, level of education, education in prehospital management, whether they have attended a 2-day separate leadership course, and whether they have experience in managing road tunnel incidents (N=20).

Descriptive data		Intervention group (n=10), n (%)	Control group (n=10), n (%)
**Sex**			
	Female	2 (20)	2 (20)
	Male	8 (80)	8 (80)
**Experience (y)**			
	5-10	4 (40)	6 (60)
	10-15	3 (30)	1 (10)
	15-20	2 (20)	0 (0)
	>20	1 (10)	3 (30)
**Level of education**			
	Registered nurse	1 (10)	0 (0)
	Specialist nurse	8 (80)	8 (80)
	EMT^a^	1 (10)	2 (20)
Education in prehospital management		9 (90)	10 (100)
Attended a 2-day separate leadership course		2 (20)	2 (20)
Have experience in managing road tunnel incidents		3 (30)	3 (30)

^a^EMT: emergency medical technician.

### Main Results

The main finding was that none (0/10, 0%) of the participants in the control group (without the tunnel-specific e-learning course) and the intervention group (with the tunnel-specific e-learning course) responded correctly to the single categorical question to enter the road tunnel. The second main finding was that the average number of correct answers among the 15 decision points was 7.0 (SD 1.8) for the control group at the first assessment point (1 month) and 6.5 (SD 1.8) for the intervention group, with no difference between the groups (*P*=.86, 2-sample test of proportions).

### Secondary Results

Tunnel-specific e-learning course exposure was not associated with more correct answers in the first or second simulation assessments when participant factors were considered in the multivariable logistic regression analysis (Table 2). Participant factors included sex, work experience, educational level, participation in a separate 2-day leadership course, and having practical experience in tunnel management. The findings showed that there were associations between more correct answers and having participated in a separate 2-day leadership course, female sex, and practical experience, at least at the first assessment point. The total response time at the first assessment point was not associated with course exposure (*P*=.80), even when considering other participant factors.

At the 6-month assessment point, 25% (2/8) of the participants in the intervention group and 14% (1/7) in the control group chose to correctly enter the road tunnel, with no difference between the groups (*P*=.59). Multivariable logistic regression analysis identified no factors associated with the total number of correct responses for either group.

Of the 20 participants, the evaluation form was completed by 11 (55%) participants (control group: n=6, 30% and intervention group: n=5, 25%). In the intervention group with 5 participants, confidence in acting as ambulance commanders was reported to be unchanged (n=3, 60%) or to have increased (n=2, 40%). In the control group with 6 participants, the confidence was reported to be unchanged (4/6, 67%) or to have increased (n=2, 33%). Regarding the course’s impact on decision-making, 3 (60%) participants in the intervention group reported it as unchanged, and 2 (40%) reported it as increased. By contrast 2 (33%) participants in the control group reported it as unchanged, and 4 (67%) reported it as increased. None of the participants reported decreased ability to make decisions or confidence in handling road tunnel incidents.

The follow-up interviews identified 3 categories linked to the decision not to enter the road tunnel correctly: *information* (lack of updated information, risk of misunderstandings, and unavailability of information on the actual tunnel system), *risks* (limited knowledge of site risks, fear of being questioned by EMS personnel if they decided to enter the tunnel, and lack of proper equipment), and *mitigation* (need of site-specific maps, access to updated course material annually, and access to aide-mémoire).

**Table 2:** Multivariable logistic regression (including demographic data, such as sex, work experience, educational level, additional leadership course, and experience with tunnel incidents), number of correct answers, and total response time at both assessment points.

Assessment point			Unstandardized β	B, 95% CI	*P* value
**Assessment point 1**					
	**Total correct answers**				
		Course exposure	–.58	–1.88 to 0.73	.36
		Sex	2.10	0.39 to 3.79	.20
		Work experience	–1.25	–2.18 to –3.25	.01
		Educational level	.75	–0.23 to 1.73	.12
		Attended a 2-day separate leadership course	3.05	0.55 to 5.55	.02
		Having practical experience in tunnel management	1.09	–0.46 to 2.64	.15
	**Total response time (s)**				
		Course exposure	44.18	–320.59 to 408.96	.80
		Sex	–216.16	–692.90 to 260.59	.35
		Work experience	–42.72	–302.35 to 216.91	.73
		Educational level	–71.07	–346.09 to 665.95	.59
		Attended a 2-day separate leadership course	–35.22	–736.39 to 665.96	.92
		Having practical experience in tunnel management	308.37	–125.75 to 742.48	.15
**Assessment point 2**					
	**Total correct answers**				
		Course exposure	–.28	–2.91 to 2.36	.82
		Sex	1.18	–3.20 to 5.57	.55
		Work experience	.30	–3.03 to 3.09	.98
		Educational level	–.57	–2.52 to 1.38	.42
		2-day separate leadership course	–3.21	–11.87 to 5.45	.52
		Practical experience in tunnel management	.98	–2.60 to 4.56	.55
	**Total response time (s)**				
		Course exposure	151.07	–236.08 to 538.21	.39
		Sex	42.21	–603.22 to 687.63	.88
		Work experience	–420.39	–870.51 to 29.74	.06
		Educational level	162.94	–123.93 to 449.80	.23
		2-day separate leadership course	1255.37	–19.22 to 2529.95	.05
		Practical experience in tunnel management	531.25	4.42 to 1058.08	.05

## Discussion

### Principal Findings

The main finding of this study was that none (0/20, 0%) of the participants correctly answered the question regarding entering the road tunnel at the first assessment point. This was unexpected.

Despite the tunnel-specific e-learning course specifically addressing how to use the unaffected tunnel tube as one of the important course learning goals, it did not succeed in supporting this expected behavior. There are several possible and different explanations for this result. One could be that most (14/20, 70%) participants lacked any experience with real tunnel incidents, and actual experience may be required to decide to enter the road tunnel properly. This is supported by other reports [45,46], which indicate that decisions often need to be based on experience. Inexperienced decision makers may depend on mnemonics and guidelines [47], and inexperienced commanders may become more hesitant in their decision-making if the guidelines or mnemonics are not adjusted to the situation or environment. Easily accessible and adequate guidelines may facilitate decision-making in this particular setting. This is supported by the participant interviews, indicating a need for adequate aide-mémoire tailored to the specific tunnel environment. Adding useful mnemonics to the tunnel-specific e-learning course may facilitate participant decision-making.

Another possible explanation for participants not actively choosing to enter the road tunnel may be concerns about personal safety. This is in line with the findings from tunnel incident reports [13,18] where incident scene safety is an important concern for the ambulance commander. In addition, it can be perceived as correct to initially wait when there is limited information about site risks. This was illustrated by the aforementioned 2013 Gudvanga tunnel fire where a large portion of the tunnel became filled with smoke due to reversed jet fans [10]. However, in the event of a fire in a twin-tube road tunnel, the unaffected tube should be regarded as a safe environment, as fans ventilate air in the same direction in both tubes, which is expected to prevent smoke spillover to the opposite tube. The unaffected tube as a safe environment was emphasized multiple times during the tunnel-specific e-learning course, which may have influenced the participants to adopt a more cautious approach. Using the opposite tube would also result in bringing the EMS personnel closer to the incident, which could be beneficial for the treatment of critical injuries, such as internal bleeding or smoke inhalation injuries. It seems that this approach needs to be addressed further in future educational efforts. For example, on-site visits could be an important element of education and provide the EMS personnel with a sense of security, which has been indicated by others [18]. Other cost-effective solutions, such as “visiting” road tunnels in a virtual environment, may create a sense of understanding of the tunnel layout.

Other possible explanations for participants choosing not to enter the road tunnel in the simulations could be related to the technical interface of the simulation medium, simulated scenarios, or the technical aspects of the assessment, which were based on MCQs. Because of this design, we could not confirm what the study participants understood in a granular manner. Participants may have considered the correct answer to the question concerning tunnel entry. There is a 20% chance of answering correctly by simply guessing. However, in this case, 0% (0/20) did, which may indicate a conscious choice to not enter the road tunnel. However, there might be challenges regarding responder clarity with MCQ formats, where the responder simply failed to convert their reasoning to an intended MCQ response. MCQs can often be improved for clarity [48,49], for example, by gathering experience with responses and modifying questions to improve precision in assessing specific reasoning [50]. Ideally, a more detailed and interactive verbal assessment could provide a more in-depth understanding of participants’ reasoning to assess their competence [51]. In this first evaluation of the simulations, MCQs were chosen to limit subjective assessor bias and facilitate participant accessibility.

Another possible explanation for not choosing to enter the road tunnel early is that the tunnel-specific e-learning course was not sufficiently clear or effective in generating specific knowledge among the participants on when to enter the road tunnel system. Although the course content was reviewed before this study, there was general agreement among the convenience-sampled stakeholders that the learning objectives were clear. It is possible that there was some knowledge decay over the weeks before the 1-month assessment. During the course, learning objectives were reinforced at multiple points. However, it is possible that the learning objectives were not adequately reinforced during the course. This result is important for the further development of this type of e-learning course, which is potentially going to be part of the formal training within the EMS system as commissioned by the Swedish Transport Administration.

Both the tunnel-specific e-learning course and the assessment were conducted in a digital environment, and the practical details of how the participants connected to the course and assessment could have influenced their learning and performance. The e-learning course and simulations used in this study were designed for use on PCs; however, how the participants accessed them was not controlled or reported. Participants’ experiences with e-learning formats, materials, or assessment methods are likely to influence learning and performance, where less familiarity and comfort with e-learning media could impede learning. A Cochrane review concluded that e-learning was no more (or less) effective than traditional learning in improving health professionals’ skills [52]. Developing this type of course or preparing for responses in complex and dynamic settings is challenging [53]. The applicability of a tunnel-specific e-learning course or SOP in a real road tunnel incident in Sweden needs to be studied further.

### Secondary Results

There was poor performance at the 6-month assessment point in both groups for the primary outcome, and the total number of correct responses did not differ between groups or between the same group at 1 month. Furthermore, secondary findings showed that participant factors, such as sex, occupational experience, and previous leadership course experience, were associated with somewhat better performance in general for the total number of correct decisions made. This can be relevant to how receptive the participants are to the tunnel-specific e-learning content. One notable secondary finding was that an additional course in leadership was associated with poorer performance in the assessment, although this was a very limited sample and may not be a true finding. It may be relevant to try to determine precourse receptivity to specific learning goals at some depth to inform course content and logistics.

Despite not observing an intervention effect in this study, participants reported that their ability to make decisions or act as ambulance commanders in road tunnel incidents increased or remained unchanged after the trial, indicating boosted or unaffected professional confidence, respectively. This personal trait (confidence), together with work experience, has been described as important for ambulance commanders in the Swedish EMS [54]. These aspects need to be included when designing this type of highly specialized e-learning course so that both experienced and inexperienced staff may benefit.

The in-depth follow-up interviews offered some insights into the reasoning behind the main outcome. In addition to the aide-mémoire, lack of information (including risks) has been identified as an issue. This is concurrent with recent findings from interviews with other emergency services than the EMS responding to road tunnel incidents [31]. A possible support tool could be an application for joint information sharing, similar to those currently used in the United Kingdom [55]. Timely access to adequate information may aid in the decision-making process and result in an informed (and quicker) decision to enter the road tunnel.

Ambulance commander performance has been assessed in different ways and reported [56] in web surveys [54] and interviews [42], with performance indicators for completing certain tasks, such as sending the first report to dispatch or formulating guidelines for response within a predetermined time frame [38-40]. Assessing performance indicators has been used as a method to evaluate incident management in Sweden [15]. This study presents a new method for evaluating ambulance commander performance using simulation-based assessment, which measures both the time and choice of decisions. With this method, each decision can be evaluated independently, potentially allowing the identification of particularly time-sensitive and difficult decisions. This type of detailed information can help to identify specific issues or points that might benefit from extra focus and resources.

### Limitations

In the context of this newly designed and tested e-learning course and the limited achievement of the learning outcomes, several study design limitations can be discussed. Despite substantial effort in validating the course content using external (EMS personnel outside the studied region) and internal (EMS personnel with in-depth training and experience in decision-making) methods, limitations in the validity and reliability of the simulations and assessment methods may have introduced systematic errors into the results. Regarding the primary outcome, the assessment method may not have detected course learning with sufficient granularity.

From a pedagogical standpoint, different methods of how study participants were presented with learning materials were used. The control group received a short lecture (passive learning), and the intervention group was active and participated in a gamified learning environment. This difference in how learning materials were provided has not been studied, as the content of the tunnel-specific e-learning course and its influence on decision-making was the main focus. However, research findings indicate that students participating in active learning environments learn more but have a lesser feeling of learning compared to students exposed to passive learning [57]. A feeling of learning less in an educational setting could have affected the participants’ decision-making abilities. In future revisions of this tunnel-specific e-learning course, the method of delivery needs to be considered. For example, using a blended learning model (including elements of both passive and active learning) may have a positive impact on learning and critical thinking [58]. In addition, more formative assessment steps in the e-learning–focused tunnel response could provide more reinforcement of primary learning goals, even though it was thought that this aspect was adequate before the study.

Using knowledge tests (MCQs) after 1 and 6 months could have influenced the result. It is known that knowledge and clinical skills decay over time [59,60]. If the first simulation had followed directly after the completion of the allotted courses, a different outcome may have been observed. This study design was chosen because there may be considerable time between an educational course and a real incident, leading to some knowledge decay. For example, major road tunnel incidents are uncommon in Sweden. During the years 2003 to 2013, a total of 926 incidents occurred in 10 of Sweden’s approximately 20 road tunnels (length of >300 m). In these incidents, 525 injuries were reported. Most (n=523) were minor injuries (injury severity score of 1-8), and no deaths were reported [61]. Hence, it may be some time before participants can apply their knowledge in a real-life setting, and retaining skills for nonroutine situations is difficult [62]. Still, none (0/20, 0%) of the participants correctly entered the road tunnel, which indicates a conscious choice. Participants were not given the correct answers after the first simulation, as this may have influenced them to change their answers in the second simulation. Further development of the e-learning course is needed to facilitate the decision to correctly enter the road tunnel in case of a major incident.

The evaluation process of the tunnel-specific e-learning course and simulation could have benefited from a more granular assessment, such as using the 4-level (reaction, learning, behavior, and results) model of assessing the effectiveness of training programs proposed by Kirkpatrick [63]. In this study, level 1 was assessed during the development phase of the tunnel-specific e-learning course. Level 3 of the Kirkpatrick evaluation model has been addressed through participants’ self-assessed responses on how their ability to make decisions or sense of security had changed. Level 2 of the Kirkpatrick evaluation model (learning) could have been addressed properly by adding a third question to the evaluation form, asking whether the participants’ knowledge of managing road tunnel incidents decreased, remained unchanged, or increased. Some aspects concerning level 2 of the Kirkpatrick evaluation model were discussed by the participants in the follow-up interviews. However, a more structured evaluation could have been used to assess learning. This element should be incorporated in future revisions of the tunnel-specific e-learning course.

Other aspects that could potentially disturb learning goal assessment in this study include external participant-specific factors or human factors, such as whether participants were unmotivated, tired, distracted, or stressed when attending the course or the simulations. Adding images, videos, and sounds to the simulation as part of realism may have also affected the participants’ focus or stress levels, for example, as information overload can degrade performance. The National Board of Medical Examiners addresses this aspect of combining media and MCQs and recommends choosing video clips with fewer distracting features [64]. Furthermore, a real road tunnel incident may include noxious sensory inputs, such as smoke and high noise levels. There must be a balance in designing training or education to incorporate enough realism without overwhelming the participant with excessive input.

Ambiguity or difficulty in interpreting the MCQs could have contributed to systematic errors in the results. More extensive work in formulating and evaluating MCQs may be an additional step in future validation processes to support both formative and normative assessment in this type of context. MCQs have the advantage of standardization and efficient data collection but, at the same time, must be carefully constructed to ensure high validity concerning specific learning goals [50]. MCQs can be useful for simple and rapid performance assessments, although other forms of assessment can provide more granular data concerning learning phenomena [51]. However, in this serious incident context, where immediate binary decisions may be needed in the setting of complex and incomplete information, MCQ assessment was judged to be similar to the rapid decision requirements in situations faced by ambulance commanders.

### Applicability

This is the first iteration of a course intended for further development based on this and other validation work for learning effectiveness in this specific, complex environment and professional response. A final e-learning course could be used to give EMS personnel a basic understanding of the tunnel environment and various risks. The final version of the e-learning course could include tunnel-specific plans and aide-mémoire and be used as a time- and cost-efficient complement to other training methods, such as tabletop or full-scale exercises. The findings in this study may be seen as a proof of concept or demonstration of feasibility for course delivery and assessment. Further evaluation and reconstruction are warranted. For example, by incorporating a simulation as the first step (and providing the user feedback on the result), the EMS personnel could be able to identify individual areas of improvement (eg, a lack of risk awareness). This feature may be useful so that personnel with different levels of experience can focus on specific sections of the course. After completing the course, EMS personnel could take a second simulation. Results from both simulations can be used to identify individual progression (or regression). Using this method, a partial individual tailoring for this kind of educational course could be constructed. Thus, the findings indicate that both the gamified e-learning content and assessments can serve as good learning alternatives. The limitations presented in this study may provide some support for other researchers considering the development of e-learning systems for content areas involving complex interventions in professional circumstances.

### Conclusions

Participation in a tunnel-specific e-learning course did not provide a measurable change in ambulance commanders’ decision-making behavior in simulated road tunnel incidents. The unwarranted reluctance of ambulance commanders in these simulations to enter the described twin-tube road tunnel system could lead to unnecessary delays in the treatment of time-sensitive injuries, with preventable morbidity and mortality, if this were to occur in a real-life situation. This type of assessment of commander performance could be applicable in a similar educational context.

## Appendix

Multimedia Appendix 1 Written information (in Swedish) sent to potential participants before enrollment.

Multimedia Appendix 2 Content outline of the tunnel-specific e-learning course.

Multimedia Appendix 3 Screenshots from the course module 3 (in Swedish).

Multimedia Appendix 4 Decisions in the simulations.

Multimedia Appendix 5 Semistructured interview guide (in Swedish).

Multimedia Appendix 6 CONSORT-eHEALTH checklist (V 1.6.1).

## Abbreviations

CONSORT-EHEALTH: Consolidated Standards of Reporting Trials of Electronic and Mobile Health Applications and Online Telehealth
EMS: emergency medical services
MCQ: multiple-choice question
METHANE: major incident, exact location, type of incident, hazards, access and egress, number of casualties, and emergency services
MIMMS: Major Incident Medical Management and Support
SOP: standard operating procedure

## Data Availability

The datasets generated or analyzed during this study are available from the corresponding author on reasonable request.
