# Supplementary material for: Ambulance Commanders’ Reluctance to Enter Road Tunnels in Simulated Incidents and the Effects of a Tunnel-Specific e-Learning Course on Decision-Making: Web-Based Randomized Controlled Trial
Source: JMIR Form Res. 2025 Mar 28;9:e58542. doi: 10.2196/58542 (PMC11992495; doi:10.2196/58542)
Supplement: Multimedia Appendix 1 [file formative_v9i1e58542_app1.pdf]

UMEÅ UNIVERSITET

Institutionen för kirurgisk och perioperativ vetenskap
Kunskapscentrum Katastrofmedicin (KcKM)
Umeå universitet, 901 87 Umeå

Information till forskningsperson avseende projektet: Utfall av utbildning angående insatspersonalens beslutseffektivitet vid simulerad vägtunnelhändelse.

Vi vill fråga dig om du vill delta i ett forskningsprojekt. Du behöver vara legitimerade sjuksköterska/specialistsjuksköterska eller ambulanssjukvårdare/undersköterska, och i din yrkesutövning kunna hamna i sjukvårdsledande position vid skadehändelse i vägtunnel. Du får inte vara diagnostiserad med post-traumatiskt stress syndrom eller egenupplevd ångest efter erfarenheter från tunnelhändelser.

Bakgrund:
Syftet med studien är att jämföra två olika utbildningars effekt på ambulanssjukvårdens ledningsfunktion (sjukvårdsledarens) förmåga att fatta beslut vid skadehändelser i vägtunnlar.

Genomförande:
Studien kommer att genomföras på distans under två sessioner. Under session I kommer du att; a) svara på frågor som handlar om deskriptiva data (exempelvis utbildningsnivå och erfarenhet) via ett videosamtal, b) randomiseras till att genomföra en utbildning (utbildning A eller utbildning B). Under session II kommer du att få göra ett muntligt test via videosamtal ungefär en månad efter genomförd utbildning. Testet kommer att genomföras vid en tidpunkt som passar dig, exempelvis utanför kontorstid. Total tidsåtgång för studien beräknas till 2,5–3 timmar. Ingen ekonomisk ersättning utbetalas för ditt deltagande.

Risker:
Vi har inte identifierat några risker med att delta i detta projekt. Deltagande i utbildningen kommer inte att innebära obehag eller smärta. Ditt deltagande är frivilligt och du kan när som helst välja att avbryta deltagandet utan att uppge varför. Om du vill avbryta ditt deltagande ska du kontakta ansvariga för projektet (se nedan).

Redovisning:
Resultatet, som innehåller avidentifierat material, kommer att redovisas i en vetenskaplig artikel, konferenser, samt i en projektrapport. Om du önskar att få en kopia av dessa skickad till dig via brev eller e-post, var god kontakta ansvariga för projektet.

Vad händer med mina uppgifter?
Studien kommer att samla in information om dig. Inspelade videosamtal och resultat från det muntliga testet kommer att förvaras enligt offentlighets- och sekretesslagen samt arkivlagen. Personuppgiftsombudet vid Umeå universitet ansvarar för dina personuppgifter. Behandlingen av dina personuppgifter sker i enlighet dataskyddsförordningen (GDPR). Enligt GDPR har du rätt att ta del av de uppgifter om dig som hanteras i studien och vid behov få eventuella fel rättade eller uppgifter raderade. Önskar du ta del av uppgifterna ska du kontakta personuppgiftsombudet **(090-786 50 00)** eller dataskyddsombudet (**pulo@umu.se**). Om du är missnöjd med hur dina personuppgifter behandlas har du rätt att ge in klagomål till Integritetsskyddsmyndigheten (tidigare Datainspektionen) som är tillsynsmyndighet.

Ansvariga för projektet:
Forskningshuvudman: Umeå universitet, 901 87, Umeå. Epost: registrator@umu.se
Ansvarig forskare: Anton Westman, läkare, med. dr. Epost: anton.westman@umu.se
Doktorand: Johan Hylander, sjuksköterska, at-läkare. Epost: johan.hylander@umu.se

UMEÅ UNIVERSITET

Institutionen för kirurgisk och perioperativ vetenskap
Kunskapscentrum Katastrofmedicin (KcKM)
Umeå universitet, 901 87 Umeå

Informerat samtycke för studien: Utfall av utbildning angående insatspersonalens beslutseffektivitet vid simulerad vägtunnelhändelse.

Jag har fått muntlig och/eller skriftlig information om studien och har haft möjlighet att ställa frågor. Jag får behålla den skriftliga informationen.

Jag samtycker till att delta i projektet: Utfall av utbildning angående insatspersonalens beslutseffektivitet vid simulerad vägtunnelhändelse.

Plats och datum	Underskrift
	Namnförtydligande
