# Supplementary material for: Ambulance Commanders’ Reluctance to Enter Road Tunnels in Simulated Incidents and the Effects of a Tunnel-Specific e-Learning Course on Decision-Making: Web-Based Randomized Controlled Trial
Source: JMIR Form Res. 2025 Mar 28;9:e58542. doi: 10.2196/58542 (PMC11992495; doi:10.2196/58542)
Supplement: Multimedia Appendix 2 [file formative_v9i1e58542_app2.docx]

| **Subject** | **Module** | **Presentation** | **Content** | **Learning Objectives** | Time (min) |
| --- | --- | --- | --- | --- | --- |
| **Tunnel course** | **1** | **Background and objectives** | | | **40** |
|  |  | **1.1** | Background and purpose  Objectives of the course | To recognize the objectives of the course |  |
|  |  | **1.2** |  |  |  |
|  | **2** | **The tunnel environment** | | |  |
|  |  | **2.1** | Type of tunnels  Security systems  Facilitation of evacuation | To understand how the tunnel system is constructed and knowledge of security systems |  |
|  |  | **2.2** |  |  |  |
|  |  | **2.3** |  |  |  |
|  | **3** | **From dispatch to arrival on-scene** | | |  |
|  |  | **3.1** | Organisational tasks before arrival Important decisions on route Location of the command post  Risk assessment  Unique risks of the tunnel environment  Decision-making and risk adherence | To understand and analyse unique risks and ability to adequately assess risk and make decisions |  |
|  |  | **3.2** |  |  |  |
|  |  | **3.3** |  |  |  |
|  |  | **3.4** |  |  |  |
|  |  | **3.5** |  |  |  |
|  |  | **3.6** |  |  |  |
|  | **4** | **The focal point of the rescue effort – finding those in need** | | |  |
|  |  | **4.1** | Methods of deciding who to treat first  Each organisations contribution  Type Injuries seen in tunnel incidents | To understand patterns of injury and evaluate each organisations contribution |  |
|  |  | **4.2** |  |  |  |
|  |  | **4.3** |  |  |  |
|  | **5** | **When the rescue effort have been concluded** | | |  |
|  |  | **5.1** | Model for structured follow-up  Mitigation and preparedness  Summary of the course | To recall methods for follow up and to understand the mitigation and preparedness processes. |  |
|  |  | **5.2** |  |  |  |
|  | **6** | **Simulation** | | | **30** |
|  |  | **6.1** | Participants are presented with a case 1 month after the course | To test one´s ability to make informed decisions |  |
|  |  | **6.2** | Participants are presented with a case 6 month after the course | To test one´s ability to make informed decisions | **30** |
