# Supplementary figures and images for: Ambulance Commanders’ Reluctance to Enter Road Tunnels in Simulated Incidents and the Effects of a Tunnel-Specific e-Learning Course on Decision-Making: Web-Based Randomized Controlled Trial
Source: JMIR Form Res. 2025 Mar 28;9:e58542. doi: 10.2196/58542 (PMC11992495; doi:10.2196/58542)

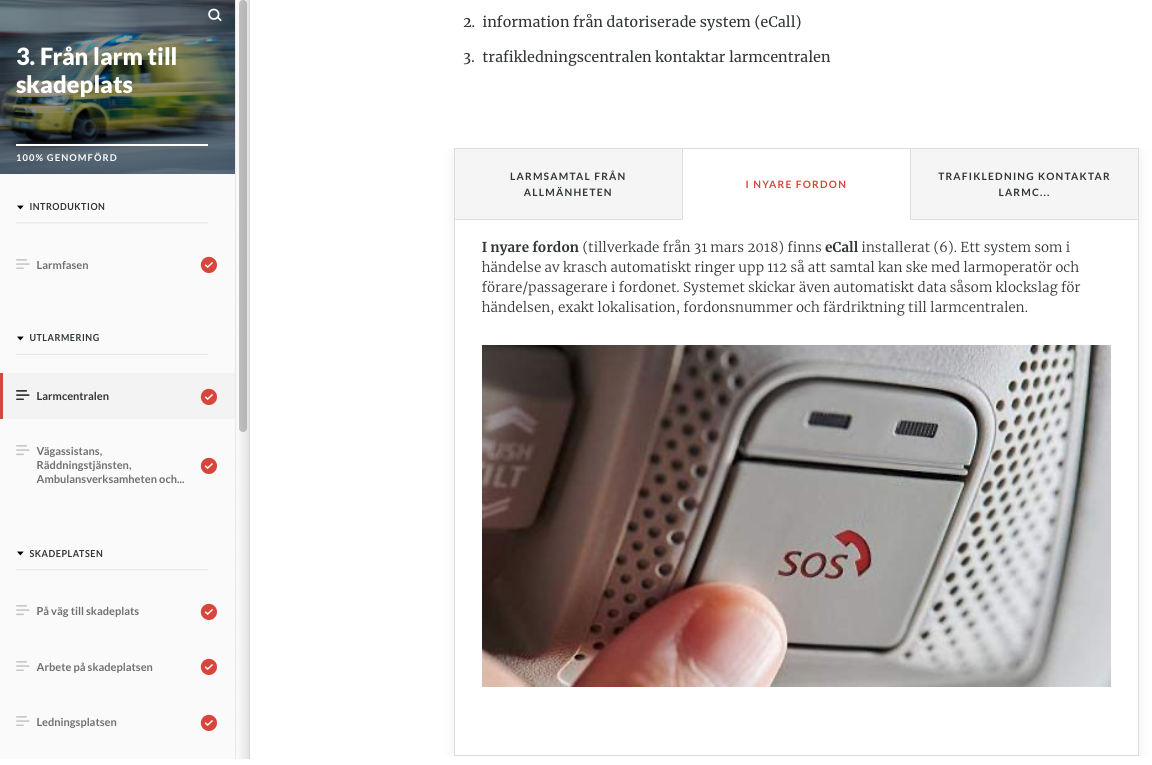


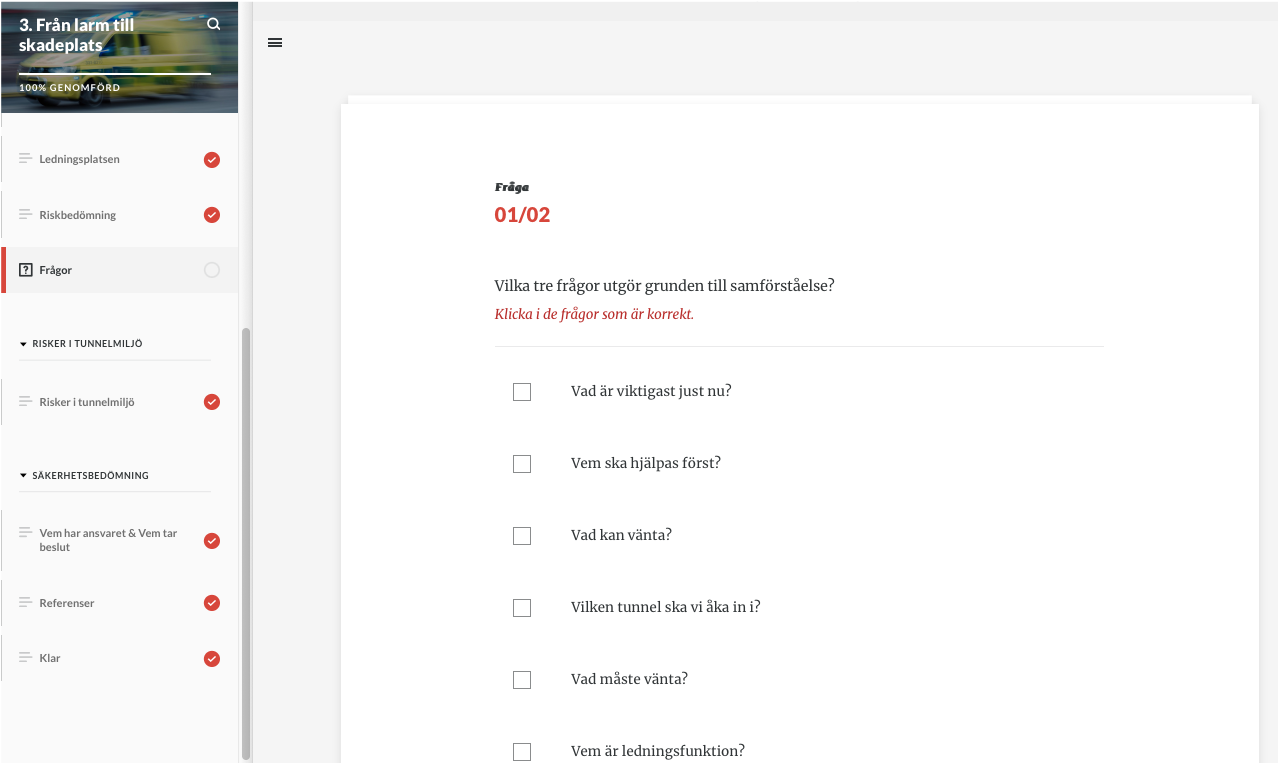

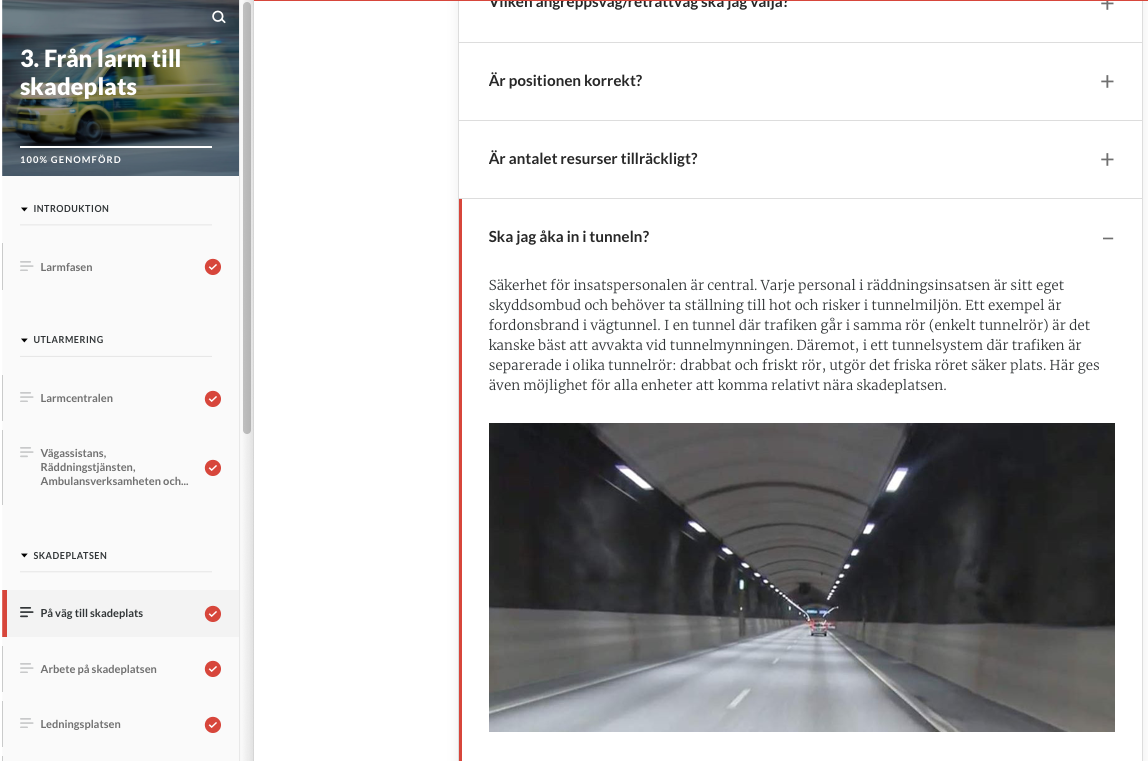

Supplement: Multimedia Appendix 3 [file formative_v9i1e58542_app3.docx]
