# Supplementary material for: Ambulance Commanders’ Reluctance to Enter Road Tunnels in Simulated Incidents and the Effects of a Tunnel-Specific e-Learning Course on Decision-Making: Web-Based Randomized Controlled Trial
Source: JMIR Form Res. 2025 Mar 28;9:e58542. doi: 10.2196/58542 (PMC11992495; doi:10.2196/58542)
Supplement: Multimedia Appendix 4 [file formative_v9i1e58542_app4.docx]

**Decision no. Content of decision**

I Suitable route to the incident site

II Asks for more information

III Location of assembly point

IV Enter the road tunnel

V First task upon arrival in the unaffected tube

VI Content of your METHANE-report

VII Second task after METHANE-report have been given

VIII Risk assessment

IX Where is it safe

X Level of care (shared decision with ambulance commander)

XI Guideline for response

XII Location of casualty clearing point

XIII Content of your second METHANE-report

XIV Which organization/s are responsible for searching the tunnel

XV Suitable egress route
