# Supplementary material for: Ambulance Commanders’ Reluctance to Enter Road Tunnels in Simulated Incidents and the Effects of a Tunnel-Specific e-Learning Course on Decision-Making: Web-Based Randomized Controlled Trial
Source: JMIR Form Res. 2025 Mar 28;9:e58542. doi: 10.2196/58542 (PMC11992495; doi:10.2196/58542)
Supplement: Multimedia Appendix 5 [file formative_v9i1e58542_app5.docx]

**Information till forskningsperson avseende projektet: Utfall av utbildning angående insatspersonalens beslutseffektivitet vid simulerad vägtunnelhändelse**. *-Preliminär intervjuguide*

**Inledning**: Muntlig verifiering av forskningspersonsinformationens innehåll. Inledande frågor kring antalet tjänsteår, utbildningsnivå och praktisk erfarenhet av insats vid skadehändelse i vägtunnel. Praktisk erfarenhet av sjukvårdsledning vid skadehändelse i vägtunnel.
**Scenario**: För en tid sedan genomförde du två simuleringar av skadehändelse i vägtunnel. Vid dessa simuleringar fick du agera i rollen som sjukvårdsledare och ta till ställning till en rad olika beslut. Ett av besluten handlade om att åka in i själva tunnelsystemet. Dagens intervju kommer att vara centrerat kring detta beslut.

**Frågor:**

*Vilka tankar dök upp när du fick frågan att åka in i vägtunneln?

*Vilka känslor upplevde du kring beslutet att åka in eller inte åka in i vägtunneln?

*Vad påverkade ditt beslut att åka eller inte åka in i tunneln?

*Hur reflekterade du kring detta beslut efter simuleringarna?

*Hur känner du för detta beslut idag, hade du tagit något annat beslut om du fick genomföra simuleringarna igen?

*Hur har ditt beslut att åka eller inte åka in i tunneln vid simuleringarna påverkat hur du skulle agera vid skarp tunnelhändelse idag?

*Hur har typen av figurerad händelse (tex brand eller krasch) påverkat ditt beslut att åka in eller inte åka in i tunneln?

*Vilka svårigheter/underlättande faktorer upplevde du kring att fatta beslutet?

*Vilka verktyg känner att du att du behöver/inte behöver för att kunna fatta beslut att åka in eller att inte åka in i en vägtunnel vid övning? Skarp händelse?

*Vilka beslutsstöd finns tillgängliga vid skadehändelse i vägtunnel?

*Eventuella följdfrågor för att få ett rikare innehåll i intervjuerna*

- Skulle du kunna utveckla mer? Vill du berätta mer?
- Hur upplevde du händelsen? Utveckla gärna.
- Har du några andra tankar/farhågor?
